# A feasibility study of two variants of a blended functional remediation programme for euthymic patients with bipolar I disorder

**DOI:** 10.1111/bjc.70000

**Published:** 2025-06-11

**Authors:** Susan Zyto, Ralph W. Kupka, Annet Nugter, Peter F. J. Schulte, Marieke van Eijkelen, Eline Regeer, Sigfried Schouws

**Affiliations:** ^1^ Department of Medical Psychology Dijklander Hospital Hoorn The Netherlands; ^2^ Mental Health Service Organization Noord‐Holland‐Noord Heerhugowaard The Netherlands; ^3^ Department of Psychiatry, Amsterdam Public Health Research Institute Amsterdam University Medical Center, Vrije Universiteit Amsterdam The Netherlands; ^4^ GGZinGeest Center for Mental Health Care Amsterdam The Netherlands; ^5^ Altrecht Institute for Mental Health Care Utrecht The Netherlands; ^6^ Plusminus (Dutch Patient Association for Bipolar Disorder) Amersfoort The Netherlands

**Keywords:** bipolar disorder, cognition, functional remediation, psychosocial functioning

## Abstract

**Background:**

Bipolar disorder (BD) is associated with reduced psychosocial functioning, partly due to cognitive impairments. Functional remediation (FR), aimed at ameliorating daily functioning, is based on psychoeducation and strategies to cope with cognitive problems. Given the limited number of studies in patients with BD, more studies are needed to evaluate different FR programmes.

**Methods:**

A total of 29 euthymic patients with BD‐I followed a 12‐session FR programme consisting of both group and individual sessions, offered in two variants: one in‐person and one online (video conferencing). Both variants were supported by E‐health modules. Feasibility was the primary outcome, as measured with dropout rates and attendance, as well as questionnaires about patients' experiences with the programme. The secondary aim was to explore effects on psychosocial functioning.

**Results:**

Results show an acceptable dropout rate. Attendance was good as 83% visited at least 10 sessions. Analyses of participants' experiences revealed gain of insight and implementation of learned strategies in daily life. Independently working with the E‐health modules did not appear feasible. Exploratory analyses showed a significant improvement in psychosocial functioning for both variants.

**Limitations:**

The results of the effect analysis are preliminary, due to a small sample and lack of a control group.

**Conclusions:**

This FR programme showed good feasibility for both the in‐person and online variant. Online treatment has advantages as it can reach out to a larger group of participants. Effect analyses indicated reduction in psychosocial impairments in both variants. Larger controlled studies are needed to investigate the treatment effects of the current FR programme.

## INTRODUCTION

Bipolar disorder (BD) is associated with marked impairments in daily life and a diminished quality of life, even in‐between mood episodes (Sylvia et al., 2017). BD can have great personal and economic consequences (Sletved et al., 2023), and in general terms, functional recovery often lags behind when compared with symptomatic recovery (Tohen et al., 2000). Impairments in various domains of functioning, such as social and work areas, persist in 30%–60% of patients with BD after symptoms have remitted (MacQueen et al., 2001; Wingo et al., 2009). Returning to the previous level of work or study is often not achieved, and occupational difficulties are common. Employment rates range from 40% to 60% (Marwaha et al., 2013) with cognitive impairments significantly contributing to occupational problems (Koene et al., 2022; Sanchez‐Moreno et al., 2009). Euthymic patients with BD experience persistent adverse effects of cognitive impairment in daily functioning both cross‐sectionally and longitudinally, independent of (residual) mood symptoms (Baune et al., 2013; Depp et al., 2012; Wingo et al., 2009). It is estimated that 50%–70% of patients with BD have significant cognitive impairments in the euthymic phase (Kjærstad et al., 2023), although there is considerable heterogeneity in the degree of impairment. The cognitive impairments occur mainly in the areas of executive functioning, attention, memory, and speed of information processing, with small to medium effect sizes compared with healthy controls (Bourne et al., 2013). Although evidence points to subgroups of patients with BD, factors such as illness duration, recurrent or more severe mood episodes, and more hospitalizations have been associated with more marked cognitive dysfunction (Van Rheenen et al., 2020). While earlier studies found that cognitive impairments were more severe in individuals with BD type I than BD type II (Simonsen et al., 2008), recent studies suggest only minimal or no difference (Jensen et al., 2021; Palazzo et al., 2017).

Rehabilitation programmes for BD have been developed in attempts to bridge the gap between symptomatic and functional recovery. Functional remediation (FR) is a treatment form that aims to restore psychosocial functioning by training the use of neurocognitive skills, with an emphasis on practical efficacy in daily life (Martinez‐Aran et al., 2011). It integrates techniques from cognitive remediation (CR) for acquired brain injury, focusing primarily on psychoeducation and compensatory strategies. Compensatory strategies are tools to mitigate the impact of cognitive impairment and comprise different techniques such as goal management training for planning, external and internal memory strategies, and tools for the management of slow speed of information processing. Additionally, FR employs techniques like modelling, role‐playing, self‐instruction, and metacognition. While CR typically incorporates methods aimed at directly restoring cognitive functions – often by computerized exercises – FR focuses on enhancing psychosocial function by the use of cognitive techniques in real‐world, ecological settings (Bellani et al., 2019; Strawbridge et al., 2021).

Studies on FR have been conducted mainly within the field of schizophrenia (Wykes et al., 2011). A review of FR in BD (Bellani et al., 2019) identified 11 studies, most of them with small groups of patients, and only three RCTs. By far the largest RCT, involving 239 outpatients, was conducted in 2013 by Torrent et al. which has set a gold standard in the field. This three‐arm study compared a 21‐week group FR programme to 21 sessions of group psychoeducation and treatment as usual (TAU). The FR programme was significantly more effective than TAU and equally effective as psychoeducation in aspects of psychosocial functioning. However, after a 1‐year follow‐up, only the FR group showed persistent improvement in the area of autonomy, compared with the other groups (Bonnin et al., 2016).

We considered a functional remediation (FR) programme of 21 sessions too long for the Dutch patient population. Therefore, in our previous pilot study of FR in patients with BD, we investigated the feasibility of a combined group and individual 12‐session programme (Zyto et al., 2016). This programme was based on psychoeducation and protocols corresponding to the recommendations for patients with BD (Martinez‐Aran et al., 2011).

We combined group and individual sessions to overcome problems with the cognitive heterogeneity among patients with BD as the individual sessions allow for a more tailor‐made approach in working towards personal goals according to the needs and capabilities of the individual patient.

The current FR programme is an adaptation intended to further intensify the treatment by adding newly developed E‐health modules with both psychoeducation and cognitive strategies. The E‐health modules were developed to bring more efficiency to the treatment as they are easily accessible. The tool can be used as an application on the smartphone and can be used to register cognitive failures and apply neurocognitive techniques when needed. The programme was originally designed as an in‐person treatment. However, because of restrictions of the COVID‐19 pandemic, lasting from 2020 to 2022, approximately, half of the patients were treated by psychologists online through video conferencing.

The objective of the current study is to test the feasibility of this newly adapted FR programme, in a group of euthymic patients with bipolar I disorder (BD‐I), with cognitive complaints, as an adjunctive intervention to TAU, and to investigate the feasibility of both the in‐person variant and the online variant. The secondary objective is to explore the effectiveness of the programme on functional outcome.

It was hypothesized that the current FR programme would be feasible in the light of the experiences with the earlier version of the programme and the readiness to follow a FR programme due to substantial psychosocial problems in patients with BD‐I. No a priori hypotheses were formulated about differences in feasibility between the in‐person and the online variant, as the online group emerged out of necessity rather than prior planning.

## METHODS

The study was performed in three specialized outpatient clinics for bipolar disorders in the Netherlands, from 2020 to 2022. Included in the study were patients meeting Mini‐International Neuropsychiatric Interview criteria for Bipolar I disorder (M.I.N.I.; Sheehan et al., 1998), aged 18–60 years, with no marked manic or depressive symptoms in the past 8 weeks. This last criterion was defined by scores <5 on the Altman Self‐Rating Mania Scale (ASRM; Altman et al., 1997) and <16 on the Quick Inventory of Depressive Symptoms‐Self Rated (QIDS‐SR; Rush et al., 2003). Patients with subjective cognitive complaints and expressing at least a moderate degree of functional impairment, as indicated by a score >18 on the Functioning Assessment Short Test (FAST; Bonnín et al., 2018), were included. Exclusion criteria were: IQ scores <85, as measured with the Dutch Adult Reading Test (DART; Schmand et al., 1998); a history of neurological disease or traumatic brain injury; and substance or alcohol abuse within the last 3 months (defined as a mean of ≥3 units/day for men, and a mean of ≥2 units/day for women), in line with Dutch Society of General Practitioners Guideline Problematic Alcohol Use (2021). We further excluded patients with ECT treatment within the last 12 months.

### Ethical considerations

The study was approved by the Medical Ethical Committee of the VU University Medical Centre, Amsterdam, the Netherlands, under number NL66487.029.18. All study participants provided written consent to participate after being given full information about the study.

### Materials and procedure

Eligible patients were screened for symptoms of depression and mania, IQ, and functional impairment, after reconfirming the diagnosis of BD‐I. At baseline (T0), quality of life and the current level of subjective cognitive complaints were measured, and participants underwent an online neuropsychological assessment. Immediately after the end of the FR programme (T1), the post‐treatment assessment included an evaluation of the programme and the user‐friendliness of the E‐health tool, followed by the functional and clinical scales as in the baseline assessment. Three months post‐treatment (T2) a follow‐up assessment was performed using the same functional and clinical scales. The in‐person variant took place in two of the three mental health centres; the online variant was given through a secure Google Meet account with participants from the third centre.

#### Intervention

We developed a FR programme that provides insight to patients with BD concerning the role of cognitive impairment in daily functioning and offers compensation for these deficits in daily life by using cognitive strategies. The programme was based on strategies for the treatment of patients with acquired brain injury (Sohlberg & Mateer, 2001) which is also recommended for BD (Martinez‐Aran et al., 2011). The content was chosen in accordance with the neuropsychological problems associated with mood disorders (Miskowiak et al., 2018; Yatham et al., 2010): memory, working memory, attention, speed of information processing, and planning. The current programme was supported by E‐health modules with information about cognitive functions and strategies. These were based on an existing programme in a booklet (‘Niet Rennen maar Plannen’, Dutch for: Don't Run but Plan; Baars‐Elsinga et al., 2013) that was modified into an online application, together with two of the original authors (Baars‐Elsinga and Visser‐Meily) and with colleagues within the field of cognition and cancer, and tested by professionals as well as persons with lived experience. After consultation with the original authors, we added some new elements aimed at work and other social situations: communication at work or similar circumstances, and strategies to be efficient in a conversation. We further added strategies for keeping attention that had been used in our earlier pilot. For homework, patients used various tools within the E‐health programme to practice with these cognitive strategies.

The 12‐session programme consists of six group sessions followed by five individual sessions and one final group session. Because of the COVID‐19 pandemic restrictions, the treatment could be administered only online through video conferencing, after approximately half of the participants had already been treated in person. Hence, there was no formal assignment to either of the in‐person or online groups. After turning to the online treatment, there were no changes to the content or procedure of the original programme.

As a consequence, the programme had two variants of which the first was offered fully in‐person on the treatment site. The second variant was offered online, through a secure Google Meet account. Between sessions, in both groups, homework assignments were made online in the E‐health application. To support the programme, a Powerpoint presentation was developed with the contents of each session. In addition, a user's manual with general instructions and the content per session guided the psychologists who delivered the programme. For each part, instructions were given together with the corresponding slide in the Powerpoint, and with the approximate time needed for each part.

The group‐based intervention consisted of six weekly sessions of two blocks of 45 minutes, with a 15‐minute break between the blocks. During the first session, cognitive problems associated with bipolar disorders and their implications for daily life were discussed in an interactive way. The subsequent group sessions consisted of specific information, homework, and exercises (for the content see Table S1).

The minimum group size was four persons and the maximum was eight persons for the in‐person condition; for the online condition, a maximum of six was considered more adequate. The intervention was administered by two licensed psychologists, trained by the coordinating investigator (SZ) who was part of the team that designed the programme.

Individual sessions 7–11:

The online protocols for cognitive strategies, which had already been introduced in the group sessions, served to work on individual goals. Participants were invited to choose a personal goal for the individual treatment and were already led to specify a concrete goal at the end of the group sessions. Initially, participants were supposed to work independently with the programme in five individual sessions, only guided by written feedback after every session through the application by one of the psychologists. However, feedback provided by the first nine participants showed that it was too demanding to make the individual sessions part of the weekly routine due to a lack of trigger or prompt. Therefore, the procedure was changed and the subsequent groups received weekly 30‐minute sessions given by one of the psychologists, in person and online, respectively.

Session 12:

One final group session to evaluate and reinforce behavioural changes made by the participants.

### Assessments

#### Sociodemographic and clinical assessment

The diagnosis of BD‐I was confirmed by the M.I.N.I. (Sheehan et al., 1998). Sections of the Questionnaire for Bipolar Illness (QBP; modified from Leverich et al., 2001) were used to gather data on sociodemographic variables, duration of illness, number of manic and depressive episodes, hospitalizations due to mania or depression, current substance or alcohol abuse, and type and dose of ongoing psychotropic medication. Depressive and manic symptoms were measured using the QIDS‐SR (Rush et al., 2003) and the ASRM (Altman et al., 1997), respectively.

#### Functional assessment

Psychosocial functioning was measured by the FAST (Rosa et al., 2007). The FAST is a structured interview consisting of 24 items containing six specific aspects of daily functioning: autonomy, professional functioning, cognitive functioning, financial functioning, interpersonal relationships, and free time. The items are scored on a four‐point scale consisting of: 0 = no problems; 1 = mild problems; 2 = moderate problems; and 3 = severe problems. The individual scores are summated to achieve a total score ranging from 0 to 72. A higher score indicates more problems in daily life. The interview was carried out by psychologists and psychological assistants, some of whom were involved in the treatment.

Health‐related quality of life was measured using the SF‐36 (Ware Jr., 2000). This 36‐item questionnaire consists of eight functional scales (vitality, physical functioning, bodily pain, general health perceptions, physical role functioning, mental health, emotional role functioning, and social role functioning). The questionnaire generates the eight‐dimensional scores as well as two summary scores for the four scales for physical health (Physical Component Summary; PCS) and the four scales for mental health (Mental Component Summary; MCS). Each scale is transformed into a scale with a minimum of 0 and a maximum of 100. A lower score indicates more disability.

#### Neuropsychological assessment

Neuropsychological tests were administered through The Amsterdam Cognition Scan (ACS, Feenstra et al., 2018). The ACS is an online, self‐administered neuropsychological assessment of seven neuropsychological tests, based on traditional neuropsychological tests. The ACS has been set up as an instrument suitable for large‐scale assessment for clinical studies. It gives a reasonable assessment of cognition when needed to define a group in a cost‐effective manner, as personnel involvement is minimal. The ACS mimics the traditional tests, but new elements were added to measure the underlying constructs. The tests measure attention, information processing speed, working memory, verbal learning and memory, visuospatial memory, executive functioning, and psychomotor speed and correspond roughly to the following traditional tests: Verbal memory: Dutch variant of Rey's Auditory Verbal Learning Task (RAVLT), 15 Words Test (Saan & Deelman, 1986); Connecting the Dots I and II: Trailmaking Test (TMT A and B; Reitan, 1958), Place the Beads: Tower of London (TOL; Culbertson & Zillmer, 1999) Fill the Grid: Grooved Pegboard (Kløve, 1963); Box Tapping; Corsi Block Test (Kessels et al., 2000). WAIS‐III digits forwards and backwards (Wechsler, 1997), Reaction Time: Visual reaction Time (FePsy; Alpherts & Aldenkamp, 1994). The correlation between the online and the traditional test scores is of medium to large concurrent validity (total score *r* = .78; Feenstra et al., 2018). Although the ACS is self‐administered, all participants were individually assessed on location at the participating sites to avoid interruption and interference at home. Scores on the neuropsychological tests were converted into *z*‐scores (mean = 0, standard deviation = 1) that were derived from the means and standard deviations of a normative population of healthy controls, differentiated by age and gender (Feenstra et al., 2018). Negative *z*‐scores indicate worse performance compared with normative data of the healthy controls.

For the experience of cognitive problems in daily life, we administered the Cognitive Failure Questionnaire (CFQ; Broadbent et al., 1982), higher scores indicating a higher level of self‐reported cognitive complaints.

#### Attendance, dropout, and compliance

The attendance and reasons for dropout were noted by the psychologists who gave the training. In addition, for the purpose of the current study, an evaluation form with open and closed‐end questions was developed to evaluate the reasons for having stopped the programme, not having attended all sessions, or not having done the homework every week.

The evaluation form was given to all participants after completion of the training, including the persons who had dropped out.

#### Participants' experiences with the programme and the software

To evaluate the experiences with the treatment and the E‐health application, the second part of the aforementioned evaluation form was used. The closed‐end questions regarding the participants' experience with the programme are rated on a semantic differential scale (range 0–6, 0 = not at all; 6 = very much). In addition, a standardized scale, The System Usability Scale (SUS; Brooke, 1996) was applied to evaluate the usability of the software. The SUS is a self‐administered 10‐item scale giving a global view of subjective assessments of usability of a system. The items are rated on a 5‐point scale ranging from 1 (I strongly agree) to 3 (I agree nor disagree) to 5 (I strongly disagree). The scores are converted into a scale ranging from 0 to 100 where a higher score implicates a higher usability. A score above 50.9 can be seen as acceptable (Bangor et al., 2009).

### Data analysis

Statistical Package for Social Sciences (SPSS) version 27 (IBM Corp., Armonk, NY, USA) was used to conduct the statistical analyses. Quantitative variables were described using the mean and standard deviation. Ordinal data were presented by median, range, or interquartile range (IQR).

To investigate objective 1 regarding the programme's feasibility, we conducted descriptive analyses. These included numbers for dropout and attendances rates, as well as medians and IQR's for the scale assessing participants' experiences. The analyses were conducted for the whole group as well as separately for the in‐person and the online group. To test the possible difference in patients' experiences between the in‐person and the online groups, we performed the non‐parametric Wilcoxon signed‐rank‐sum test, due to non‐normally distributed data and small sample sizes. In addition, this test was used to compare the two groups on relevant demographic and clinical variables. The open‐ended questions of the participants' experiences were analysed through open and axial coding and finally selective coding, by two of the researchers (SZ and ME) independently. This method, derived from grounded theory (Glaser & Strauss, 1967), allows for analysing qualitative data. In the phase of open coding, the texts were coded according to the meaning, and in the axial coding phase, interconnected underlying themes were identified. In the last phase, overarching themes were selected. Differences in all steps were discussed until consensus was reached.

To investigate objective 2 of the study, the treatment's effectiveness, we conducted the non‐parametric Wilcoxon signed‐rank test to analyse possible differences between the scores on T0 and T1, respectively T0 and T2 for the whole group. Likewise, we added an analysis with the Wilcoxon signed rank‐sum test to explore the differences between the in‐person group and the online group on psychosocial functioning at the same timepoints. Missing values in the questionnaires were replaced through mean imputation. If more than 10% of the values were missing in an individual questionnaire, it was discarded from further analysis. The alpha value was set to .05. Effect sizes were calculated with Cohen's *d*. The threshold of >1 SD below the mean was used to define cognitive impairment (Miskowiak et al., 2018).

## RESULTS

### Clinical and sociodemographic characteristics

**FIGURE 1:**
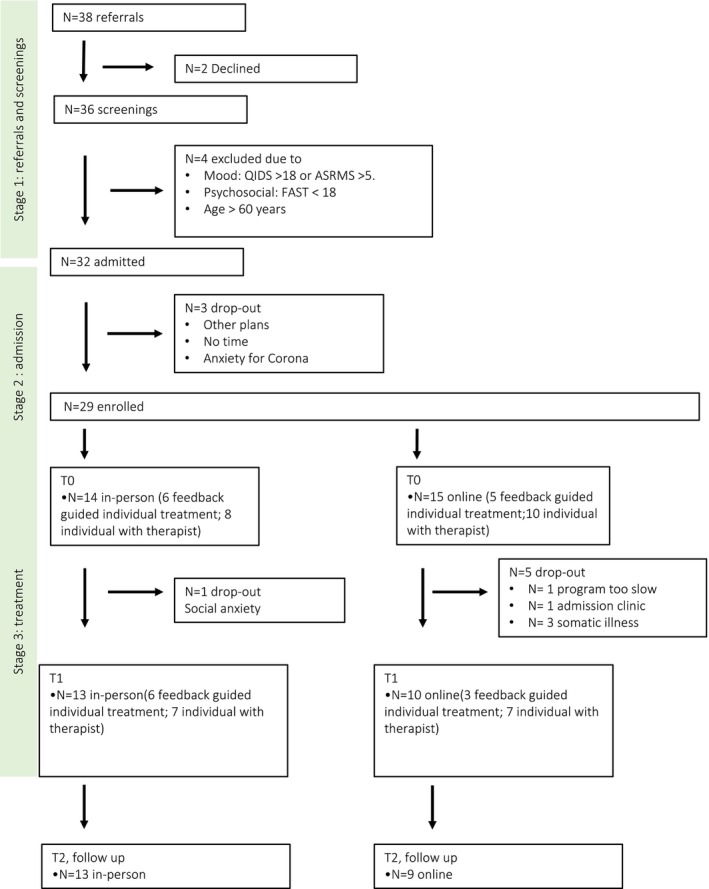
Flowchart.

Of the 38 patients who were referred for participation, 32 patients were admitted to the programme: two patients declined and four referred patients did not fulfil inclusion criteria. Of these 32, three patients dropped out before the programme started. The reasons for these early dropouts differed: one patient feared contracting COVID; one patient chose to go on holiday; and one patient expressed not having enough time. A total of 29 patients started the FR programme, 14 followed the in‐person variant and 15 the online variant. In Figure 1 the flow of the patients throughout the study is visualized.

The clinical and sociodemographic variables of all patients, and the in‐person and online groups separately, are summarized in Table 1. At study entry, patients were euthymic with mild residual depressive symptoms and very low symptoms of (hypo)mania. The average estimated IQ was 106.9 (SD 10.2). Most participants had completed a higher vocational training. The overall psychosocial functioning was on average at least moderately impaired (FAST mean = 26.3; SD 8.0). Most participants had substantial disabilities at work; 15 were on permanent sick leave, of whom six worked on a voluntary basis, 10 were working part‐time, two were reintegrating after sick leave, and two persons worked full‐time, of which one had adjusted work according to his current abilities.

**TABLE 1:** Demographic and clinical characteristics^a^.

Variables	Whole group *N* = 29, mean (SD)	In‐person *n* = 14, mean (SD)	Online *n* = 15, mean (SD)
Age	46.7 (11.0)	50.2 (9.0)	43.4 (12.0)
Gender			
Female, number (%)	*n* = 19 (64.7)	*n* = 9 (64.3%)	*n* = 10 (66.7%)
Male, number (%)	*n* = 10 (35.3)	*n* = 5 (35.7%)	*n* = 5 (33.3%)
Educational level^b^, Mdn, (min–max)	6 (3–7)	6 (3–7)	6 (5–7)
Estimated IQ (DART)	106.9 (10.2)	104.5 (11.5)	109.2 (8.7)
Duration of illness (years)	26.4 (12.8)	31.6 (12.4)	22.6 (12.4)
Manic episodes (number)	9.3 (6.7)	9.1 (6.8)	9.4 (6.9)
Depressive episodes (number)	10.1 (7.7)	8.4 (7.4)	11.8 (7.8)
Hospitalizations (number)	1.3 (1.9)	1.8 (2.1)	.8 (1.4)
Depressive symptoms (QIDS‐SR)	9.5 (4.8)	8.6 (4.8)	9.6 (5.1)
Manic symptoms (ASRM)	1.4 (1.8)	1.4 (1.8)	1.4 (2.3)
Psychosocial functioning (FAST)	26.3 (8.0)	26.8 (7.0)	26.0 (9.3)
SF‐36			
PCS	70.4 (18.4)	65.3 (19.9)	75.5 (16.1)
MCS	62.05 (22.5)	60.3 (18.9)	63.8 (26.3)
Work functioning^c^ (QBP), *Mdn* (min–max)	3 (0–4)	2.3 (0–4)	2.8 (1–2)
CFQ	54.7 (11.9)	55.3 (9.9)	52.5 (13.4)

Abbreviations: ASRM, Altman Self‐Rating Mania Scale; CFQ, Cognitive Failure Questionnaire; DART, Dutch Adult Reading Test; FAST, Functioning Assessment Short Test, higher scores indicate more problems; MCS, Mental Component Summary; Mdn, median; QBP, the Questionnaire for Bipolar Illness; QIDS‐SR, Quick Inventory of Depressive Symptomatology‐Self‐Rated; PCS, Physical Component Summary; SF‐36, 36‐item short form health survey.

^a^ No significant between‐group differences were found (all *p* > .05) for age, gender, education, IQ, manic symptoms, depressive symptoms, and psychosocial functioning, based on Wilcoxon rank‐sum tests and Chi‐square tests (gender). Exact *p*‐values are reported below.

^b^ Education is based on Dutch education scores 1–7 (Verhage, 1964), corresponding with the following US years of education: 1: 1–5 years, 2: 6 years, 3: 7–8 years, 4: 7–9 years, 5: 7–10 years, 6: 7–16 years, and 7: 17–20 years.

^c^ No disability = 0; mild disabilities = 1; moderate disabilities = 2; substantial disabilities = 3; severe disabilities = 4.

Comparisons between the in‐person group (*n* = 14) and the online group (*n* = 15) showed no significant differences on relevant demographic and clinical variables: age (*z* −1.01; *p* = .32), gender (*x* ^2^ = .9); IQ (*z* −1.53; *p* = .13); education (*z* −2.63; *p* = .53); psychosocial functioning (*z* −.35; .75); depression (*z* −.79 *p* = .45) or (hypo)manic symptoms (*z* −.24; *p* = .8).

### Neuropsychological assessment

The means, SD and *z*‐scores of the neuropsychological tests for the whole group are summarized in Table S2. The cognitive impairment of the participants was characterized by a slower reaction time than in the normative group of healthy controls (mean *z* = ‐1.02). Direct learning and working memory ability were at the level of low average compared to healthy controls (mean *z* = ‐0.64). Performances of verbal memory recognition, planning and non‐verbal memory span were comparable to healthy controls.

### Attendance and dropout

The first 11 participants (six in‐person; five online) of the FR programme followed only a feedback‐guided variant of the individual programme. After the procedure changed, as described previously, the subsequent participants were seen for 30‐minute sessions with a psychologist who had administered the group programme, in‐person or online (if participating in the online version). Of these 29 participants, five persons dropped out of the online condition, and one from the in‐person condition (see Figure 1). Two dropped out because of physical health issues, not related to BD, and one person due to health issues in the family. One person became hypomanic. One person in the online condition felt the programme was too slow and found it annoying to listen to the other participants. One person dropped out of the in‐person condition due to social anxiety.

A total of 23 participants completed the programme, fully or partly (see Figure 1).

Due to dropouts shortly before the first session, one in‐person group started with only two participants while originally having included five.

In general, the six group sessions were well attended. The average group attendance was 5.2 (1.0) sessions, range 3–6, median 6 for the in‐person condition (*n* = 13) and 5.7 (.5), range 1–6, median 6, for the online condition (*n* = 10).

The average attendance of the six individual sessions for the feedback‐guided in‐person variant was 3.0 (3.3) (*n* = 6, of which *n* = 3 did not attend any individual sessions) and for the feedback‐guided online variant 6.0 (.0) (*n* = 3). The three participants who did not follow any session gave as a reason that it was too difficult due to a lack of reminder to work with the programme, and they also found it too difficult to work alone. The following patients were treated in individual sessions given by a psychologist, either in‐person or online. After implementing this change, the average attendance in the in‐person group was 5.6 (1.1) (*n* = 7) and 6.0 (.0) (*n* = 7) in the online variant.

Twelve participants followed all 12 sessions, while another seven persons completed at least 10 sessions in total.

### Participants' experiences

On the question how the participants had experienced the programme, the median was 5 (ranging from 0: not fruitful to 6: very fruitful) for both groups (in‐person *n* = 13; online *n* = 9). On the question if the participant had gained new insights, the median was 5 (ranging from 0: no new insights to 6: very many new insights) for both groups. For total time that had been invested in the programme, the median was 3 (ranging from 0: precisely good to 6: much too much) for the in‐person group and 4 for the online group. The median of the question about user‐friendliness of the E‐health application was 4 (ranging from 0: very bad to 6: very good) for both groups. The average scaled sum of the SUS scale was 59.1 (SD 22.6) for the whole group, indicating sufficient acceptability of the E‐health application.

The further questions concerned the experiences with the group and individual sessions separately. Table 2 describes the median for the questions about patients' experiences with the group sessions for the in‐person condition and the online condition separately.

**TABLE 2:** Participants' experiences with group programme *n* = 22 (scale 0–6; 0 = very negative; 6 = very positive).

Experiences	In‐person *n* = 13, Mdn	Online *n* = 9, Mdn	*z*‐Score^a^	*p*‐Value
*Very dissatisfied/very satisfied*	4.7	5.0	−.31	.75
*Very demanding/very easy*	4.3	2.9	−2.09	.037*
*Dislike/like*	4.9	4.0	−1.26	.21
*Not worth it at all/very worth it*	4.7	4.6	−.11	.92
*Not pleasant/very pleasant*	4.8	4.1	−1.04	.36

Abbreviation: Mdn, Median.

^a^ Wilcoxon rank‐sum test.

* *p* < .05.

Analyses show that the groups differed significantly on how demanding group sessions were considered, with lower scores for the online condition.

Because of the shift in procedure concerning the individual sessions, from feedback guided to face‐to‐face sessions, the data for the individual sessions are divided into four sections, concerning the condition in‐person/online and if the sessions were feedback guided/face‐to‐face; see Table 3.

**TABLE 3:** Participants' experiences with the individual programme *n* = 21 (scale 0–6; 0 = very negative; 6 = very positive).

Experiences	In‐person feedback^a^ *n* = 5, Mdn	In‐person sessions^b^ *n* = 7, Mdn	Online feedback^a^ *n* = 3, Mdn	Online sessions^b^ *n* = 6, Mdn
*Very dissatisfied/very satisfied*	4.0	5.0	4.0	5.0
*Very demanding/very easy*	3.0	4.0	3.0	4.0
*Dislike/like*	3.0	5.0	3.0	5.0
*Not worth it at all/very worth it*	4.0	6.0	4.0	5.0
*Not pleasant/very pleasant*	4.0	5.0	4.0	4.5

Abbreviation: Mdn, median.

^a^ Feedback in the E‐health application from a psychologist after completion of a task.

^b^ 30‐minute sessions with a psychologist.

Participants seem to evaluate the individual sessions with a psychologist as more satisfying than working with the feedback in the E‐health application. No tests were conducted due to the small size of the groups.

### Open‐ended questions

Table 4 presents the themes and subthemes that emerged from the analyses of the open‐ended questions, for the whole group, concerning the benefits of the intervention and on which areas the largest changes had taken place. The analyses resulted in themes related to the psychoeducation and strategies and to more personal issues concerning awareness and insight. The largest changes were reported in the areas of having grip on life and in self‐awareness and acceptance. Implementing strategies had also led to task‐specific improvements such as reading a book or being able to concentrate on various tasks.

**TABLE 4:** Qualitative analysis of open‐ended questions *n* = 22.

Questions	Themes	Subthemes
What did you benefit from the most	*Strategies and theory*	Planning Keeping attention Theory of memory To set a goal, aim Mnemonics
	*Sharing experience with group*	Insight and awareness Tips to tackle things Stories to learn from Broader vision Recognition
On which areas did you notice the largest changes	*Personal change*	Better grip on my life Better grip on my time Self‐awareness and acceptance I don't get overstimulated anymore Awareness of pitfalls
	*Tasks Functioning*	Keeping attention at the task Reading is possible again Concentrated working To set goals and work out in planning No double dates anymore

Feedback derived from the question ‘what would you like to change about the programme’ most often concerned the online application. It was reported that the E‐health application did not connect clearly with the group sessions, because it had much more content than was used during the session. This made it somewhat difficult to navigate and find specific tools and homework. Nevertheless, the user‐friendliness of the online application itself was rated as satisfactory.

### Treatment effect analyses

**FIGURE 2:**
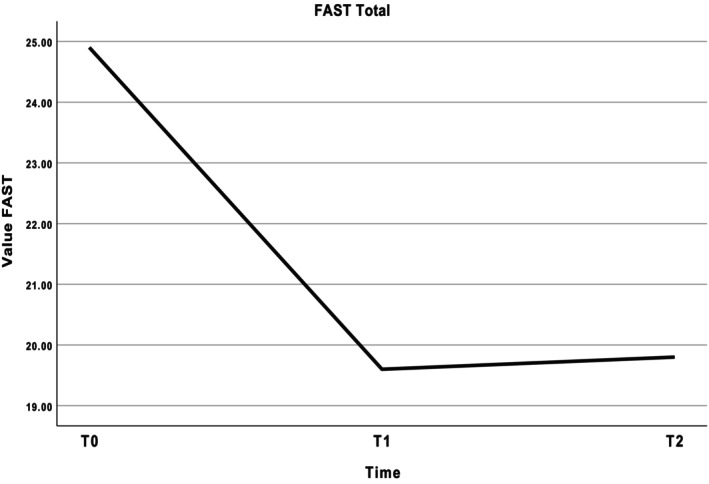
FAST total mean scores at T0 = baseline (*n* = 17), T1 = immediately after intervention (*n* = 16), T2 = at 3‐month follow‐up (*n* = 18). FAST, Functioning Assessment Short Test; higher scores indicate greater impairment.

**FIGURE 3:**
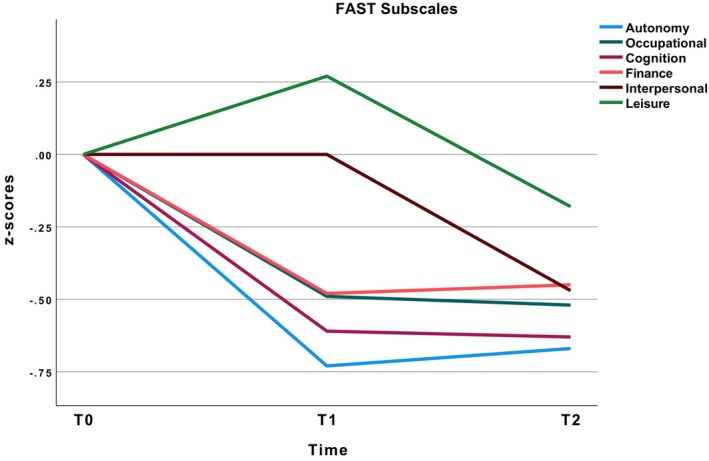
*Z*‐scores of the FAST subscales at T0 = baseline (*n* = 17), T1 = immediately after intervention (*n* = 16), T2 = at 3‐month follow‐up (*n* = 18). FAST, Functioning Assessment Short Test; higher scores indicate greater impairment.

The results of the treatment effect analyses for the whole group are given in Table 5. A significant treatment effect was found for the FAST total score directly after the end of the intervention (Cohens' *d* .60) while the effect was stronger during the follow‐up at 3 months (Cohens' *d* .88), see Figure 2. Subscale analyses of the FAST show the subscales ‘autonomy’, ‘occupational functioning’, and ‘cognition’ to be significantly reduced after the intervention and still at follow‐up, indicating less problems in these areas (see Figure 3). On the other scales, depression, (hypo)‐mania, quality of life, and subjective cognitive functioning no statistically significant differences were found between T0 and T1 and T0 and T2 respectively.

**TABLE 5:** Treatment effect analyses, whole group, from T0 to T2.

Measures	T0 *n* = 19, Mdn (IQR)	T1 *n* = 17, Mdn (IQR)	T2 *n* = 18, Mdn (IQR)	Comparison^a^ T0–T1		Comparison^a^ T0–T2	
				*z*‐Score	*p*‐Value	*z*‐Score	*p*‐Value
FAST	26.0 (11.0)	18.0 (17.0)	16.5 (17.3)	−3.14	.002**	−3.62	<.001***
FAST_aut	2.5 (3.3)	1.0 (2.0)	.5 (2.0)	−2.83	.005**	−2.46	.01*
FAST_occ	12.5 (9.3)	9.0 (10.5)	9.5 (13)	−2.17	.03*	−2.25	.02*
FAST_cog	6.5 (4.0)	3.0 (2.5)	3.5 (3.5)	−2.69	.007**	−3.03	.002**
FAST_fin	1.5 (2.3)	.0 (1.5)	.0 (2.0)	−1.03	.30	−1.51	.13
FAST_int	4.0 (4.0)	4.0 (4.0)	3.0 (4.0)	−.12	.91	−1.88	.06
FAST_ lei	1.0 (1.3)	1.0 (2.0)	0 (2.0)	−.40	.69	−.55	.58
SF‐36							
PCS	75.6 (32.3)	80.6 (27.5)	73.8 (37.5)	−1.14	.26	−.40	.69
MCS	61.3 (40.1)	69.6 (22.3)	68.6 (38.6)	−1.70	.88	−.23	.82
QIDS‐SR	9.0 (7.0)	6.0 (4.5)	7.5 (6.75)	−1.63	.10	−1.86	.06
ASRM	.0 (2.0)	1.0 (3.0)	1.0 (3.0)	−1.83	.07	−1.30	.20
CFQ	50.5 (19.5)	43.0 (22.0)	43.5 (19.0)	−.88	.38	−1.30	.20

Abbreviations: ASRM, Altman Self‐Rating Mania Scale; CFQ, Cognitive Failure Questionnaire; FAST, Functioning Assessment Short Test; FAST_aut, autonomy; FAST_cog, cognition; FAST_fin, finance; FAST_int, interpersonal relations; FAST_lei, leisure time; FAST_occ, occupational functioning; IQR, interquartile range; MCS, Mental Component Summary; Mdn, Median; PCS, Physical Component Summary; QIDS‐SR, Quick Inventory of Depressive Symptomatology‐Self Rated; SF‐36.

^a^ Wilcoxon signed‐rank test.

* *p* < .05.

** *p* < .01.

*** *p* < .001.

#### Between groups comparisons

When comparing the total FAST score between the in‐person variant and the online variant, no statistically significant differences were found (see Table S3). We limited the analysis to the total FAST score because the groups were small and examining differences between the groups was not an initial topic of interest.

## DISCUSSION

The aim of this pilot study was to investigate the feasibility of a FR programme with two variants, one in‐person and one online via video conferencing, for euthymic patients diagnosed with bipolar I disorder and having subjective cognitive complaints. Secondarily, we aimed to explore the treatment effects of this FR programme.

The analyses of dropouts, as a measure of feasibility, showed a dropout rate of 21% for the whole group. In the FR study of Torrent et al. (2013) involving 21 sessions, the dropout rate was around 29%. In our study, more persons dropped out of the online variant, with one participant stopping because of an overall low satisfaction with the programme. The other dropouts from the online variant seem unrelated to the treatment as such. On the other hand, more participants of the in‐person variant followed less than 10 sessions. Of the participants that completed the programme, 83% attended at least 10 of the 12 sessions, which can be seen as satisfactory. Compared with the in‐person variant, the participants of the online variant found the group sessions to be more demanding to follow. Furthermore, feedback revealed that working independently without a therapist during the individual sessions was not easily implemented in daily life due to lack of prompts and difficulties working alone. Subsequently, shifting to face‐to‐face sessions improved compliance. While feedback‐guided online therapy is as effective in the treatment of depression as standard treatment with individual cognitive behavioral therapy (CBT) (Cuijpers et al., 2019), working independently, with only feedback through the online application, might be too difficult for individuals with BD having cognitive problems. However, since only the goals and the choice for the online module were set beforehand, it might be necessary to give more prompts and set up a more structured plan for working independently online, and focus more on establishing a good therapeutic alliance.

As to the further experiences, qualitative analyses revealed that participants often found the strategies for planning useful and had implemented these in their daily lives. Also, participants reported they had gained insight and acceptance during the treatment. Feedback on points of improvement concerned how the accompanying online application was connected to the sessions. It was seen as rather difficult to navigate in the online modules since these did not directly correspond to the group sessions as only parts of the modules were used for the psychoeducation and homework. Still, the user‐friendliness of the online application itself was rated as satisfactory.

Exploratory analyses of treatment effects revealed a significant reduction of psychosocial impairment after the intervention, with a medium effect after the intervention and a large effect at the 3‐month follow‐up. The higher effect size at follow‐up may suggest that participants needed more time to implement the acquired skills. These effects are in line with the 21‐session intervention by Torrent et al. (2013). Further analyses of the subscales showed a significant reduction of impairment in the areas of autonomy, occupational functioning, and cognition. Both our previous pilot study (Zyto et al., 2016) and the RCT by Torrent et al. (2013) showed improvement in the area of occupational functioning, among others. Further analyses did not reveal any statistical difference between the in‐person group and the online group regarding the outcome of psychosocial functioning. Although the groups are too small to draw firm conclusions, this may suggest that the effects are the same for both variants. This occurred even though the participants of the online variant rated the group sessions as more demanding.

Of the clinical scales, symptoms of depression and (hypo‐) mania remained stable at all time points. These findings suggest that the reduction of psychosocial impairment is not due to the improvement of mood.

The participants typically had a high vocational training and were moderately impaired in psychosocial functioning with severe disabilities in occupational functioning. Neuropsychological assessments revealed low average scores in the area of memory and working memory, and a slower reaction time than average. These scores are lower than expected according to the level of formal education and correspond well to the averages of BD known from the literature (Miskowiak et al., 2018). Many factors other than neuropsychological impairments can interact and contribute to reduced psychosocial functioning in BD, such as residual mood symptoms, co‐morbidities, both psychiatric and somatic (Van Rheenen et al., 2020). Nevertheless, even when cognitive functioning is not objectively in the impaired range, subtle cognitive problems can be an obstacle while performing a skilled occupation. Given the severe work disabilities and loss of jobs that had occurred already, we argue that FR should take place much earlier in the course of the illness.

Many participants had not only implemented the strategies for planning in their daily lives but also reported that they had a greater sense of control over their lives. Planning skills are a key component of the executive functions, which are higher order processes involved in regulating behaviour and emotions (Diamond, 2013). Effectively planning daily activities requires multiple skills, including scheduling appointments, estimating time for a task, maintaining focus, and inhibiting distractions. Additionally, planning techniques help prevent cognitive overload by balancing daily activities and breaking down the steps of a task. It would be interesting to explore whether the self‐regulation gained through planning strategies could lead to improved resilience against mood episodes in the long term.

An important clinical implication of the findings is that the FR programme for patients with BD‐I is feasible in daily practice and may be effective in reducing psychosocial impairments, both as an in‐person treatment as well as a fully online treatment. The programme may be implemented rather well next to standard treatment of these patients and it is advisable not to wait too long to offer the programme. An important precondition for the feasibility of the programme is that the individual sessions are guided by a therapist. Online sessions may be too demanding to follow in the absence of professional guidance; however, clearer instructions and prompts might facilitate working independently.

Our study has several limitations, related to the nature and primary aims of this feasibility study. First, it concerns a relatively small study group. Moreover, during the study, there were several changes in the study procedure, partly due to COVID‐19 restrictions, so that the groups to be compared became even smaller. Second, we did not include a control group, and the secondary outcome analyses of treatment effects therefore are only exploratory. Moreover, the interviews were not always carried out by independent raters. In addition, the groups were not randomized and they were too small to rule out that the higher amount of dropouts from the online variant were not random, but an effect of the way the treatment was offered.

Furthermore, the study was divided over three centres with different therapists. This gives additional feasibility as it shows that it is possible for newly trained therapists to carry out the treatment in slightly different settings. But at the same time, this can be seen as a limitation for the measurement of treatment effects as the validity would have profited from less variance in the administration of the programme.

A strength of the current study is the innovation of giving a feasible online alternative. Although there is already evidence, from the area of psychotherapeutic treatment for mood disorders, that the efficacy of treatment through video onferencing is equivalent to that of in‐person treatments (Papola et al., 2023), whether individuals with cognitive impairments are able to follow a treatment online is not that obvious. To the best of our knowledge, this is the first FR programme that was also delivered through video conferencing. Providing treatment online has many advantages. First, it can be made available to many patients no matter their place of residence. Moreover, not having to travel is beneficial for any patient following a 12‐week programme. Second, the expertise can be concentrated in a few centres so patients are not dependent on whether their particular mental health service provides an FR programme. Groups can fill up more easily as participants can be spread out to the available treatment groups and do not need to wait too long for a group to start in a particular location. A further advantage of the current programme is that it is relatively short but offers depth into the personal functioning through the individual sessions.

In conclusion, this FR intervention, with both in‐person and online video conferencing variants for euthymic patients with BD, showed a good feasibility given the acceptable dropout rate and satisfying attendance. Participants' experiences revealed more acceptance and insight into their cognitive problems, next to the implementation of acquired strategies in daily life, also after the intervention. Exploratory analyses of treatment effects showed a reduction in self‐reported psychosocial impairments for both groups. Though our study showed a good feasibility and promising effects, a larger‐scale RCT is needed to investigate the effects of the current FR programme.

## AUTHOR CONTRIBUTIONS

**Susan Zyto:** Conceptualization; methodology; formal analysis; writing – original draft; investigation; project administration. **Ralph W. Kupka:** Conceptualization; methodology; writing – review and editing. **Annet Nugter:** Validation; writing – review and editing. **Peter F. J. Schulte:** Conceptualization; methodology; writing – review and editing. **Marieke van Eijkelen:** Formal analysis; writing – review and editing. **Eline Regeer:** Writing – review and editing; investigation. **Sigfried Schouws:** Conceptualization; methodology; investigation; writing – review and editing.

## CONFLICT OF INTEREST STATEMENT

The authors declare no conflict of interest.

## Supporting information


Table S1.–S3.

## Data Availability

The data that support the findings of this study are available from the corresponding author upon reasonable request.
