# Supplementary material for: A feasibility study of two variants of a blended functional remediation programme for euthymic patients with bipolar I disorder
Source: Br J Clin Psychol. 2025 Jun 11;65(1):15–32. doi: 10.1111/bjc.70000 (PMC12889212; doi:10.1111/bjc.70000)
Supplement: Supplementary file 1 — Table S1.–S3. [file BJC-65-15-s001.docx]

**Table S1.** Content of group sessions

| Group Session Theme | Content |
| --- | --- |
| 1. Welcome and Introduction | Theory of neuropsychological functioning in mood disorders. Sharing examples of cognitive problems and solutions.  Homework: Register what and when you forget |
| 2. Memory | Discussion of homework: Sharing experiences with situations of forgetfulness. Theory of memory functioning, internal and external strategies.  Homework: Practice memory strategies in different situations. |
| 3. Attention/communication | Discussion of homework: Sharing use of memory strategies. Theory of working memory and attention. Strategies to cope with attention problems. Strategies for conversations.  Homework: Practice strategies for keeping attention. |
| 4. Planning | Discussion of homework: Sharing experiences with attention strategies.  Theory of goal setting, motivation and strategies of planning.  Homework: Practice strategies of planning |
| 5. Speed of Processing | Discussion of homework: Sharing experiences with planning.  Theory of slowing of information processing, “Time Pressure Management”.  Homework: Practice strategies for managing time |
| 6. Communication at work/preparation individual sessions | Discussion of homework: Sharing examples of time pressure management.  Theory of communication at work and sharing experiences.  Evaluation, and goalsetting for the individual treatment  Homework: Decide on a goal for the individual sessions. |

**Table S2.** Neuropsychological assessment n=28†

| Domain/test | Mean(SD) | Mean z-score |
| --- | --- | --- |
| Attention  *-digits span forwards*  *-Connecting the Dots I* | *8.9(1.7)*  *70.2(25.0)* | *-0.65*  *-0.91* |
| information processing  -*speed/reaction speed(s)* | *354.5 (92.8)* | *-1.01* |
| working memory  *-digits span backward*  *-Box Tapping^e^ (number correct)* | *6.9(2.4)*  *9.3(2.1)* | *-0.67*  *0.00* |
| verbal memory 15 words test  *- direct learning*  *- delayed*  *-recognition* | *46.8(8.9)*  *10.4(2.9)*  *44.32(1.2)* | *-0.61*  *-0.41*  *0.09* |
| executive functioning  *-Place the Beads (total sets)* | *25.9(14.0)* | *0.06* |
| psychomotor speed  *-Connectiong the Dots II (s)*  *-Fill the Grid (s)* | *40.0(14.5)*  *56.7 (32.2)* | *-1.03*  *-0.62* |

*†in one participant no neuropsychological assessment was performed*

**Tabe S3.** Differences between groups at T0, T1 and T2.

| Timepoints |  | FAST_in-person  Mdn (IQR) | FAST_online  Mdn (IQR) |  | z-score | p-value |
| --- | --- | --- | --- | --- | --- | --- |
| T0  T1  T2 |  | 26.0(12.3), n=12  20.5(19.5), n=12  19.5 (21.3),n=12 | 25.0(10.0),n=7  18.0(11.5),n=5  14.0(15.0),n+6 |  | -1.46  -0.91  -1.27 | 0.14  0.36  0.20 |

Abbreviations: Mdn=Median; IQR=interquartile range; FAST= Functioning Assessment Short Test.

*Note:* No significant between-group differences were found (all p > 0.05) based on Wilcoxon rank-sum tests.
